# Bibliographical Notices

**Published:** 1844-10-05

**Authors:** 


### BIBLIOGRAPHICAL NOTICES.

### FIRST PUBLICATIONS OF THE SYDENHAM SOCIETY.

The Sydenham Society, instituted 1843. *The Epidemics of the Middle Ages.* From the German of J. F. C. Hecker, M. D., Professor at Frederick William's University at Berlin, and member of various learned Societies in Albany, Berlin, Bonn, Copenhagen, Dijon, Dresden, Erlangen, Hanau, Heidelberg, Leipzig, London, Lyons, Marseilles, Metz, Naples, New York, Offenburg, Philadelphia, Stockholm, Toulouse, Warsaw and Zurich. Translated by B. G. Babington, M. D., F. R. S., &c. 8vo. pp. 418. London, 1844.

The Sydenham Society, instituted 1843. *Researches on Phthisis, anatomical, pathological and therapeutical.* By P. C. A. Louis, M. D., Physician to the Hôtel Dieu; Perpetual President of the Medical Society of Observation; Member of the Royal Academy of Medicine; Honorary Member of the Medical Societies of Massachusetts and of Edinburgh, of the Provincial Medical and Surgical Association of England; Fellow of the College of Physicians and of the Medical Society of Philadelphia; of the Royal Academy of St. Petersburg; of the Medical Societies of Heidelberg and

Bruges; of the Medical Society of Observation of Boston. 2d edition, considerably enlarged. Translated by Walter Hayle Walshe, M. D., Professor of Pathological Anatomy in University College, London; Physician to the Hospital for Consumption and Diseases of the Chest; Member of the Medical Society of Observation of Paris, &c. 8vo. pp. 571. London, 1844.

The two first works published by the Sydenham Society are now in the hands of the subscribers in this country; the third and last for 1843, a complete Latin edition of the works of Sydenham, is on its way hither. The whole cost of one year's subscription, will be *between six and seven dollars*; and any one of the works is nearly worth the money;—we mean of course, commercially.

The Council assert, that they have devoted nearly the whole income to the bare expenses of editing, printing and binding, and will place in the hands of the members three handsome volumes, comprising about 2000 pages of close print, amounting in value to at least double that of the subscription.

The society has met with unusual success. At the annual meeting held on the first of May—Sir James Clark in the chair—it was stated, that the number of members then enrolled amounted to 1700; and that continual accessions were taking place. At the present day, the number cannot be far, if at all, short of 2000. It was the original intention of the Council to issue the works of Sydenham in the original Latin, as a natural commencement of their operations; but this intention they were compelled to forego. "A good Latin edition of Sydenham," say they, "has long been a desideratum; and it has been felt by all scholars to be a disgrace, that the country, which boasts of having given birth to a second Hippocrates, should never yet have produced a complete edition of his works, worthy of their author. In appointing Dr. Greenhill of Oxford as the editor of Sydenham's works, the Council feel assured that this desideratum will be supplied; and they are happy in being able to report, that the Latin edition will in the course of a few weeks be in the hands of the subscribers. The amount of labour required, and the pains which Dr. Greenhill has bestowed on his task, are the sole causes that have prevented the earlier issue of Sydenham's works."

"In reviewing the past year and the present state of the Sydenham Society," the Council add, "it must be admitted to have succeeded far beyond the most sanguine expectations of its founders; nor can it fail to be a source of well founded rejoicing to all who have the interests of the profession at heart, that so large a number of its members have given their support to an institution having for its object, not the gratification of any mere temporary appetite, or the supply of any ephemeral or trifling wants, but of those lasting stores of solid information and improvement, whence not only the present but future generations must derive permanent advantage. It augurs well for the future welfare of the profession, that so many of its junior members are to be found in the first list of our subscribers. In conclusion, the Council have to express their thanks for the kind and patient indulgence that has hitherto been accorded them. In getting the necessary machinery of so large and important a Society into steady and regular operation, many difficulties naturally occurred, and much time was requisite. Having, however, now a number of books in preparation, the Council trust, that they will be enabled to present the subscribers, at short intervals of time, with works which, from their own nature, as well as from the character it is wished the society should maintain, have necessarily demanded much time and labour in editing. The full amount of benefit that the Society is capable of conferring on its members, cannot, however, be ensured, except by the continual efforts of each one to increase its numbers. It is only by this means that any hope can be entertained of extending the plan of its operations, so as to fulfil the desires expressed by many of its present supporters. It is therefore earnestly hoped that all who are anxious for its success, will exert themselves to the utmost in making known its objects, and in increasing its members."

It may be well to add the following note appended to the "Regulations for the delivery of books" in the Report of the Second General Meeting of the Society.

"*Nearly the whole of the impression of* 1750 *of the three works for the first year being now appropriated, the Council cannot promise to supply any new subscribers for the past year. Those gentlemen, however, who may wish to have the first year's publications, are requested to inform the Secretary, when their subscriptions for the second year are paid. The names of such persons will be registered in the order in which they are sent in, and if any copies remain of the first year's publications, they will be appropriated in the same order. If, however, the number of those who have expressed a desire for the first year's publications, shall at any time be sufficient to justify it, a reprint of those works will be determined on.*"

The subscription constituting a member is *five dollars*, to be paid in advance on the 25th day of March annually, for which he is entitled to a copy of every work published by the Society for the year for which he subscribes. "Any subscriber who shall not have paid his subscription previous to the last day in July of the current year, shall not be allowed the privileges of that year's subscription *except on the payment of a fine of five shillings; nor shall any one be allowed to join the society for the current year after that period, except on payment of a similar fine.*"

The fine will not be enforced on this side the Atlantic; but it must be borne in mind, that delay may prevent the subscriber from obtaining the books which he desires, as the whole edition may be disposed of before he makes up his mind,

Thus much for the course and prospects of the Society. As regards the works issued by them.

1. The treatises of Hecker furnish interesting information in regard to some of the most terrific epidemics of which history makes mention;—which appeared and disappeared, like the cholera of modern periods, without any plausible reasons having been suggested to explain their coming or their going. The copy right of the Black Death was presented to the Society by Dr. Babington; that of the Dancing Mania was negotiated for by him, and both were prepared by him for the press, together with a translation, now for the first time made public, of the Sweating Sickness. As an appendix to the last, is added Caius's *Boke or Counsell against the Disease commonly called the Sweate or Sweatyng Sicknesse,*" of which two copies only are known to exist;—one in the British Museum, and one in the Library of the College of Physicians of London.

That which has happened once may occur again. It is fortunate, however, that for the production of these fearful epidemics, the precise combination of causes that produced them but rarely occurs; still the catenation may take place; and then works like that of Hecker become invaluable. Under any circumstance, indeed, they are replete with interest to the learned physician, as they are to the statesman and philanthropist.

2. The second work—that of Louis—treats of a disease, which instead of visiting us at immense intervals of time, is rife—fearfully rife—in almost every portion of the world. It, therefore, must be acceptable to all; and the Council feel rightly when they say they "are persuaded, that they have only acted in accordance with the grand design of the Society in placing this work in the hands of nearly 2000 of their professional brethren." It is the second edition of the work of the great observer; and one of the productions which first gave him the elevated character he enjoys wherever his name is known. The translator, Dr. Walshe, was a pupil of M. Louis, and is in every way qualified for the task.

It is but necessary to add, that the two works, in execution and general appearance, may take their place on the shelves of the Library, along with the best productions of the British bookcraft.

---

*The Medical Student, or Aids to the study of Medicine. A revised and modified edition.* By Robley Dunglison, M. D.; Professor of the Institutes of Medicine, &c. &c. 12 mo. pp. 311. Philadelphia: Lea and Blanchard, 1844.

The first edition of this work appeared some years since. It originated, we are told, in the numerous applications made to the author for his opinion, as to the best method of study for one about to enter upon professional life, as well as for one engaged in its prosecution. Another edition being called for by the publishers, the author has embraced the opportunity to subject the work to an entire revision.

It now consists of four chapters, on the following subjects: Chapt. 1. On "Preliminary education:" or the amount and kind of learning required to qualify a student for entering upon the study of medicine.

Chapter 2. "Medical education prior to attendance on lectures."

Chapter 3. "Medical education during the period of attendance on lectures."

Chapter 4. "Medical education after graduation."

In the course of his discussions on the various topics embraced in these chapters, (and which comprehend a great variety) the author's observations are designed to apply exclusively "to the study of medicine as taught in this country." He "has entered into few or no speculations as to what medical education ought to be. The work is intended simply as some guide to the American medical student, who, too frequently, is totally uninformed as to the course he ought to pursue—not only when he commences to read professional subjects, but before he enters a medical college for the prosecution of his studies there."

Beside the author's general qualifications for writing a work like the present, his great experience as a teacher, and much intercourse with students, have served to make him particularly acquainted with the difficulties they generally experience in their progress. Accordingly, we find in this production many useful suggestions and valuable hints. The proper method and objects of study, are clearly pointed out; designating such as are important from those which are merely convenient or expedient. It will be admitted, we think, by all who read this publication, that it contains much curious and instructive matter, and is well calculated to guide the student and young physician in the successful prosecution of their studies.

---

*Anatomical Atlas, illustrative of the structure of the human body.* By Henry H. Smith, M. D., Fellow of the College of Physicians, Member of the Philadelphia Medical Society, &c., Under the supervision of William E. Horner, M. D., Professor of Anatomy in the University of Pennsylvania, &c., Part V. Philadelphia, Lea and Blanchard, 1844.

This number is occupied with "*the Nervous System and the Senses*," and contains "*one hundred and thirty-six figures*," and an *index* of the illustrations of the whole work.

The present P"art" completes this valuable work, and is in all respects equal to those which have preceded it.

In noticing the several "*parts*" as they successively appeared, we found occasion to express our decided commendation both of the plan and its execution; and now that the work is completed, we can only affirm of the *whole* what we have already said of the "*parts*"—it is a performance which reflects great credit upon *the author, the artists*, and *the publishers*. In a few instances we thought it our duty to point out what appeared to us to be errors, but of no great moment; and once, (in reference to Fig. 432 and 433) we fell into error ourselves. The fact that the work has been carried through the press under the supervision of Professor Horner, is a sufficient guarantee for its general accuracy.

